# Immune cells and high-density lipoprotein cholesterol derivative markers mediate the impact of hypertriglyceridemia on hyperuricemia in diabetes mellitus

**DOI:** 10.3389/fimmu.2026.1816335

**Published:** 2026-05-05

**Authors:** Wei Wang, Xiu Ping Qiu, Yang Chen, Xiu Li Guo

**Affiliations:** National Metabolic Management Center, Longyan First Affiliated Hospital of Fujian Medical University, Longyan, Fujian, China

**Keywords:** hypertriglyceridemia, hyperuricemia, inflammation and oxidative stress, lymphocyte to high-density lipoprotein cholesterol ratio, monocyte to high-density lipoprotein cholesterol ratio

## Abstract

**Background:**

As markers of inflammation and oxidative stress, immune cells and high-density lipoprotein cholesterol (HDL-c) derivative markers, such as the monocyte to HDL-c ratio (MHR) and lymphocyte to HDL-c ratio (LHR), have been widely utilized in clinical practice. This study aims to elucidate the roles of MHR and LHR in the impact of hypertriglyceridemia (HTG) on hyperuricemia (HUA) in diabetes mellitus (DM) from a clinical perspective.

**Methods:**

The final analysis included 976 individuals with DM. The correlations of MHR and LHR with HTG and HUA were evaluated using the restricted cubic splines (RCS) and binomial logistic regression analysis. Additionally, the mediating role of MHR and LHR in the effect of HTG on HUA was assessed using an adjusted mediation analysis.

**Results:**

Participants in the HTG and HUA groups demonstrated significantly elevated MHR and LHR levels (All *P < 0.001*). After applying adjustment for potential confounders, MHR remained a significant association with HTG and HUA, with *ORs (95% CI)* of 1.59 (1.43-1.77) for HTG and 1.13 (1.04-1.23) for HUA (both *P < 0.05*). Similarly, LHR exhibited independent correlations with HTG and HUA, yielding *ORs (95% CI)* of 2.34 (1.91-2.88) for HTG and 1.63 (1.33-2.00) for HUA. In the RCS analysis, MHR exhibited a nonlinear relationship with both HTG (*P for nonlinear = 0.001*) and HUA (*P for nonlinear < 0.001*). Conversely, LHR demonstrated a linear correlation with HTG (*P for nonlinear = 0.062*) and HUA (*P for nonlinear = 0.268*). Furthermore, the adjusted mediation analysis revealed that both MHR and LHR partially mediated the effect of HTG on HUA, accounting for the mediated proportion of 20.51% and 37.17%, respectively.

**Conclusion:**

Both MHR and LHR partially mediate the effect of HTG on HUA, indicating the vital effect of inflammation and oxidative stress in this association.

## Introduction

Hyperuricemia (HUA) is a prevalent metabolic disorder that has seen a significant rise in recent years, largely attributed to increasing rates of unhealthy lifestyles. It serves as the primary pathological and physiological trigger for gouty arthritis due to the abnormal purine metabolism (1). In addition to its association with gouty arthritis, elevated uric acid (UA) levels that exceed saturation can result in the formation of urate crystals in the kidneys and blood vessels. This condition is recognized as a main contributor to hypertension (2), chronic kidney disease (3), and cardiovascular and cerebrovascular disorders (4). As a systemic disease affecting multiple organ systems, HUA is highly prevalent among individuals with diabetes mellitus (5). Addressing HUA from a pathophysiological perspective will enhance both treatment and prevention strategies, ultimately improving the management of this complex disorder in diabetes mellitus (DM). UA is not merely a product of the purine metabolism. Increasing evidence has demonstrated that UA also plays a multifaceted role in inflammation and oxidative stress (6, 7). Elevated UA levels may facilitate the repair of infections and injuries by stimulating the immune system and promoting inflammatory responses. Additionally, UA scavenges free radicals, thus protecting cells and tissues from oxidative damage. This protective mechanism may help mitigate the effects of chronic diseases. Hypertriglyceridemia (HTG) represents one of the most prevalent lipid abnormalities encountered in clinical practice with elevated triglycerides (TG). This condition is associated with various health issues, such as pancreatitis (8) and cardiovascular disease (9). Moreover, HTG is frequently observed in patients with HUA, suggesting a significant association between these two conditions (10–12). Increasing evidence indicates that HTG may promote the development of atherosclerosis by exacerbating inflammation and oxidative stress (13). However, it remains unclear whether HTG influences HUA through similar mechanisms.

The pathophysiology of HUA is characterized by a pronounced inflammatory response and oxidative stress, and the dysregulation of immune cells (such as monocytes, macrophages, and lymphocytes) acts as the key mediator of the inflammatory processes associated with elevated UA levels (14), significantly contributing to tissue damage and the progression of related comorbidities (15, 16). High-density lipoprotein cholesterol (HDL-c) has been widely demonstrated as a protective lipoprotein for the cardiovascular system. Recent research has further elucidated that HDL-c is involved in several critical functions, including reverse cholesterol transfer, antioxidant and anti-inflammatory effects, as well as the maintenance of endothelial function (17). The interplay between immune cells and HDL-c within the context of inflammation and oxidative stress has facilitated the generation of derived indices, such as the monocyte to HDL-c ratio (MHR) and lymphocyte to HDL-c ratio (LHR). These indices integrate both factors and demonstrate a significant capacity to reflect levels of inflammation and oxidative stress. Moreover, emerging evidence supports the association of MHR and LHR with cardiometabolic diseases (18, 19), underscoring their potential as biomarkers for assessing the impact of HTG on HUA. This study aims to explore the roles of the MHR and LHR in the relationship between HTG and HUA from a clinical perspective, using mediation analysis in a cohort of DM, thereby enhancing our understanding of the contributions of inflammation and oxidative stress within this complex interplay.

## Study design and methods

### Study population enrollment

This cross-sectional study recruited participants admitted to the inpatient Endocrinology department who provided informed consent to participate in the national metabolic management center (MMC) initiative from March 2022 to December 2024. The MMC is designed to provide comprehensive management for the diagnosis and treatment of metabolic disorders, with particular emphasis on obesity and DM. It employs a comprehensive strategy for screening for complications and uses a standardized, intelligent diagnostic and therapeutic platform to ensure rigorous quality control, thereby facilitating the collection of accurate and reliable data. The study was approved by the ethics committee according to the Declaration of Helsinki (IC-2022-009). Participants were eligible for inclusion based on the following criteria: 1) fulfillment of the classification and diagnostic criteria for DM, including type 2 diabetes mellitus (T2DM), type 2 diabetes mellitus, special type of DM, gestational diabetes mellitus (20); and 2) availability of complete data. Individuals presenting with one of the following conditions were excluded from the study: 1) Accompanied by infectious disease (acute or chronic), autoimmune diseases, anemia, critical illness, malnutrition, hemolytic diseases, leukemia, or other bleeding disorders that could affect serum monocyte and lymphocyte counts; 2) malignant tumors, severe renal dysfunction (stages 4-5), severe heart failure, or the use of medications such as diuretics and glucocorticoids that could influence serum UA levels; and 3) chronic liver disease, hypothyroidism, or acute or chronic pancreatitis that could impact serum TG levels. Consequently, a total of 1,503 participants were screened; 527 were excluded based on the specified criteria, leaving 976 participants for the final analysis (**Supplementary Figure 1**).

### Study covariates and mediators assessment

The study covariates included a range of demographic, clinical, and laboratory parameters: age, diabetic duration, sex, alcohol consumption (yes or no), smoking status (yes or no), hypertension status (yes or no), body mass index (BMI), waist circumference (WC), total cholesterol (TC), low-density lipoprotein cholesterol (LDL-c), alanine aminotransferase (ALT), aspartate aminotransferase (AST), albumin, blood urea nitrogen (BUN), creatinine, glycated hemoglobin (HbA1c), and homeostasis model assessment of insulin resistance (HOMA-IR). MHR and LHR served as the mediators, calculated using the following formula: MHR = Monocytes (10^8^/L)/HDL-c (mmol/L); LHR = Lymphocytes (10^9^/L)/HDL-c (mmol/L); HOMA-IR = fasting serum insulin (µU/ml) x fasting plasma glucose (mmol/l)/22.5. Smoking was defined as participants who smoked continuously or accumulated more than four cigarettes per week for at least six months. Alcohol consumption was defined as participants who consumed alcoholic beverages more than once a year.

Demographic and anthropometric data were collected by the trained MMC researchers. After fasting for more than 12 hours, blood samples were carefully collected by the research nurses and subsequently sent to the laboratory for analysis. The auto-biochemical analyzers were employed to measure the biochemical indices. The high-performance liquid chromatography was used to determine the HbA1c level. The Coulter LH 780 analyzers were employed to calculate the monocyte and lymphocyte count.

### Exposure and outcome variable assessment

HTG was designated as the exposure variable, while HUA served as the outcome variable. Participants were classified as having HTG if their fasting TG levels exceeded 1.7 mmol/L (21). HUA was defined as fasting UA levels that surpassed 420 µmol/L for both men and women, confirmed by measurements taken on two non-consecutive days under normal dietary conditions (22). Serum TG and UA levels were determined by the auto-biochemical analyzers (Roche Diagnostics Corporation).

### Statistical analysis

A comparative analysis of baseline characteristics categorized by HTG and HUA statuses was conducted by the independent samples t-test or chi-squared (χ²) tests as appropriate. The correlations of MHR and LHR with HTG and HUA were evaluated using the binomial logistic regression analysis after adjusting for confounding variables in three models: Model 1 was adjusted for age, diabetic duration, sex, alcohol consumption (yes or no), smoking status (yes or no), hypertension status (yes or no), BMI, and WC. Model 2 further incorporated adjustments for HbA1c, TC, LDL-c, ALT, AST, albumin, BUN, creatinine, and HOMA-IR, building upon Model 1. Model 3^a^ included further adjustment for HUA based on Model 2, while Model 3^b^ adjusted for HTG based on Model 2. Additionally, the nonlinear association between MHR, LHR, HTG, and HUA was performed using the restricted cubic splines (RCS) analysis. Moreover, the threshold effects of MHR and LHR on HTG and HUA were analyzed using a two-piecewise logistic regression model. The two-piecewise logistic regression model is superior to the single linear logistic regression model when the log-likelihood ratio is less than 0.05. The mediating effects of MHR and LHR in the impact of HTG on HUA were evaluated by an adjusted mediation analysis based on bootstrapping 1,000 samples. Finally, sensitivity analyses were performed employing previous diagnostic criteria for HUA (23) (UA > 420 µmol/L for men and > 360 µmol/L for women) to further confirm the findings. A *P-value* < 0.05 (two-tailed) was deemed statistically significant. The SPSS (version 23.0) and R language software (version 4.2.3) were employed to analyze and manage the data.

## Results

### Baseline characteristics based on HTG and HUA statuses

**Figure 1:**
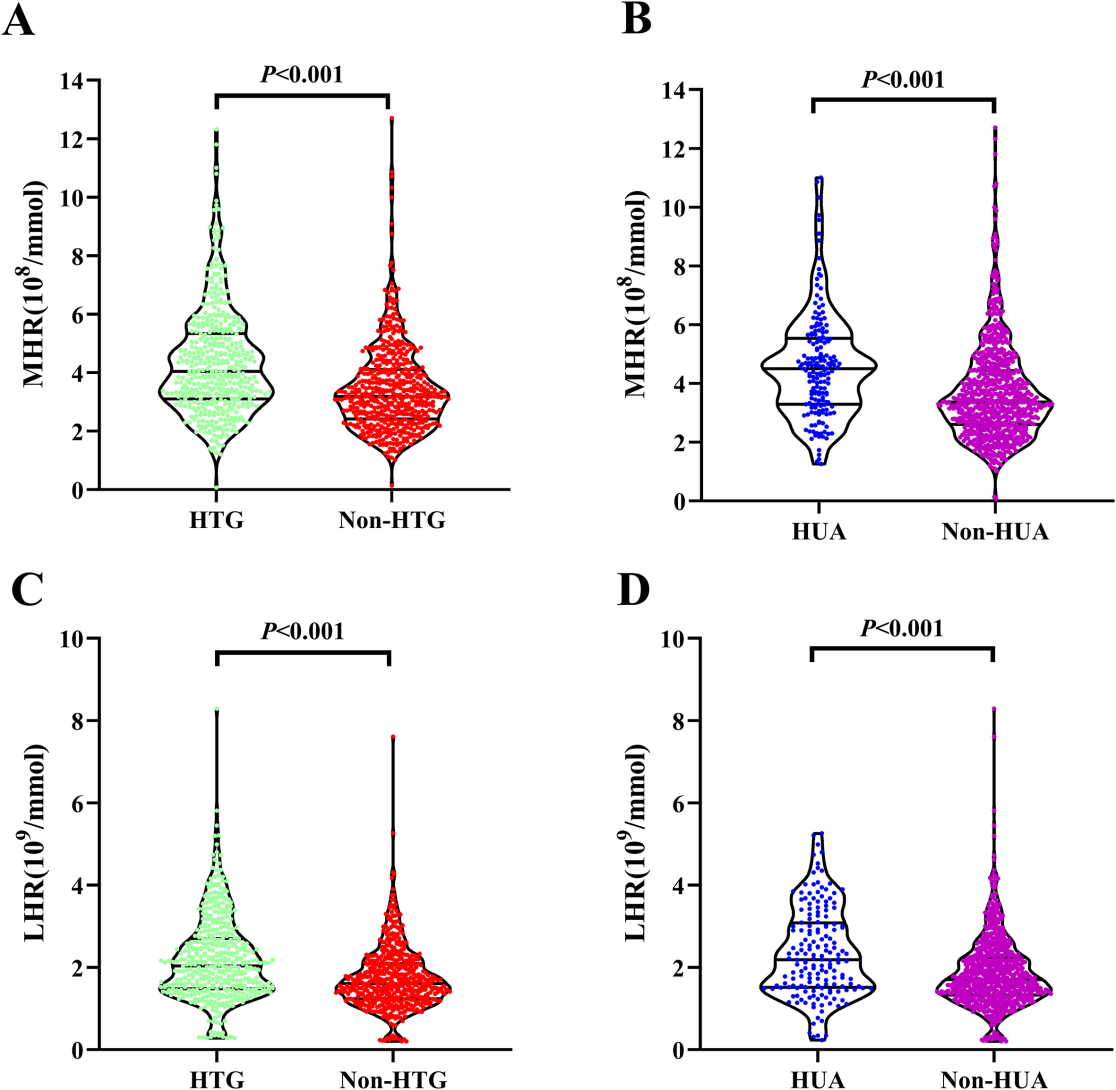
Distribution of MHR **(A, B)** and LHR **(C, D)** based on the statuses of HTG and HUA. Data was analyzed by the independent samples t-test. MHR, monocytes to high-density lipoprotein cholesterol ratio; LHR, lymphocytes to high-density lipoprotein cholesterol ratio; HTG, hypertriglyceridemia; HUA, hyperuricemia.

A total of 976 participants with DM were enrolled in this study. Among these, 440 individuals (45.1%) exhibited HTG, and 172 individuals (17.6%) demonstrated HUA, with a mean age of 54.5 ± 10.9 years. **Table 1** summarizes a comparative analysis of baseline characteristics categorized by HTG and HUA statuses. Participants with both HTG and HUA were older and exhibited significantly elevated levels of BMI, WC, TG, UA, ALT, AST, HOMA-IR, as well as monocyte counts (All *P < 0.05*). Conversely, levels of HDL-c were significantly reduced in these groups (all *P < 0.05*). Additionally, as illustrated in **Figure 1**, participants in the HTG and HUA groups demonstrated markedly higher levels of MHR and LHR (All *P < 0.001*).

**Table 1:** Baseline characteristics of study population based on HTG and HUA statues.

Characteristic	HTG			HUA		
	With (n=440)	Without (n=536)	*P value*	With (n=172)	Without (n=804)	*P value*
Age (year)	53.1 ± 11.5	55.8 ± 10.1	<0.001	55.4 ± 8.2	53.5 ± 8.1	<0.001
Diabetic duration (year)	6.4 ± 6.1	7.0 ± 6.3	0.146	6.2 ± 6.3	6.9 ± 6.2	0.227
BMI (kg/m^2^)	25.2 ± 3.8	23.8 ± 3.5	<0.001	25.4 ± 3.6	24.2 ± 3.7	<0.001
WC (cm)	91.4 ± 9.9	87.0 ± 9.8	<0.001	92.1 ± 9.6	88.3 ± 10.1	<0.001
HbA1c (%)	9.88 ± 2.37	9.50 ± 2.51	0.016	9.51 ± 2.3	9.71 ± 2.49	0.175
TG (mmol/L)	3.18 ± 1.81	1.17 ± 0.32	<0.001	2.67 ± 2.06	1.95 ± 1.45	<0.001
TC (mmol/L)	5.47 ± 1.41	4.94 ± 1.25	<0.001	5.23 ± 1.50	5.17 ± 1.32	0.561
HDL-c (mmol/L)	1.02 ± 0.27	1.23 ± 0.29	<0.001	1.07 ± 0.33	1.15 ± 0.29	0.003
LDL-c (mmol/L)	3.47 ± 0.97	3.16 ± 0.95	<0.001	3.31 ± 1.01	3.30 ± 0.96	0.860
UA (umol/L)	362.5 ± 102.3	320.4 ± 88.0	<0.001	491.7 ± 61.1	306.8 ± 63.8	<0.001
ALT (IU/L)	30.4 ± 21.3	25.6 ± 19.1	<0.001	32.3 ± 25.0	26.7 ± 18.9	<0.001
AST (IU/L)	26.7 ± 18.7	23.1 ± 13.1	<0.001	28.4 ± 18.6	24.0 ± 14.9	<0.001
Albumin (g/L)	41.3 ± 4.1	40.5 ± 4.3	0.001	41.2 ± 4.0	40.8 ± 4.3	0.187
BUN (mmol/L)	5.71 ± 1.95	5.90 ± 3.23	0.278	6.51 ± 2.31	5.67 ± 2.79	<0.001
Creatinine (umol/L)	71.9 ± 23.8	70.0 ± 24.9	0.215	86.9 ± 32.9	67.5 ± 20.6	<0.001
HOMA-IR	6.21 ± 5.63	4.13 ± 4.06	<0.001	5.44 ± 5.92	4.99 ± 4.70	0.277
Monocytes (10^8^/L)	4.18 ± 1.59	4.02 ± 1.40	0.015	4.62 ± 1.33	4.01 ± 1.50	<0.001
Lymphocytes (10^9^/L)	2.11 ± 0.78	2.00 ± 0.64	0.025	2.37 ± 0.93	1.98 ± 0.63	<0.001
sex, n (%)						
Men	272 (61.8)	335(62.5)	0.827	137 (79.7)	470 (58.5)	<0.001
Women	168 (38.2)	201 (37.5)		35 (20.3)	334 (41.5)	
Smoking, n (%)						
With	171 (38.9)	200 (37.3)	0.620	70 (40.7)	302 (37.4)	0.424
Without	269 (61.1)	336 (62.7)		102 (59.3)	503 (62.6)	
Alcohol consumption, n (%)						
With	175 (39.8)	207 (38.6)	0.713	79 (45.9)	303 (37.7)	0.044
Without	265 (60.2)	329 (61.4)		93 (54.1)	501 (62.3)	
Hypertension, n (%)						
With	164 (37.3)	198 (36.9)	0.915	65 (37.8)	297 (36.9)	0.834
Without	276 (62.7)	338 (63.1)		107 (62.2)	507 (63.1)	

Data are expressed as n (%) or mean ± SD. BMI, body mass index; WC, waist circumference; HbA1c, glycated hemoglobin; TG, triglyceride; TC, total cholesterol; HDL-c, high-density lipoprotein cholesterol; LDL-c, low-density lipoprotein cholesterol; UA, uric acid; ALT, alanine aminotransferase; AST, aspartate aminotransferase; BUN, blood urea nitrogen; HOMR-IR, homeostasis model assessment insulin resistance; HTG, hypertriglyceridemia; HUA, hyperuricemia.

### Correlation of MHR with the risk of HTG and HUA

**Figure 2:**
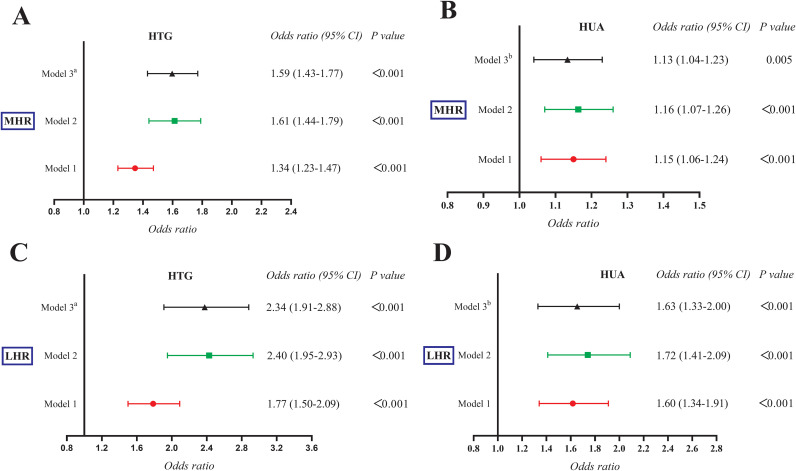
The correlations of MHR **(A, B)** and LHR **(C, D)** with HTG and HUA after adjustment in three models using the binomial logistic regression analysis. MHR, monocytes to high-density lipoprotein cholesterol ratio; LHR, lymphocytes to high-density lipoprotein cholesterol ratio; HTG, hypertriglyceridemia; HUA, hyperuricemia.

**Figure 3:**
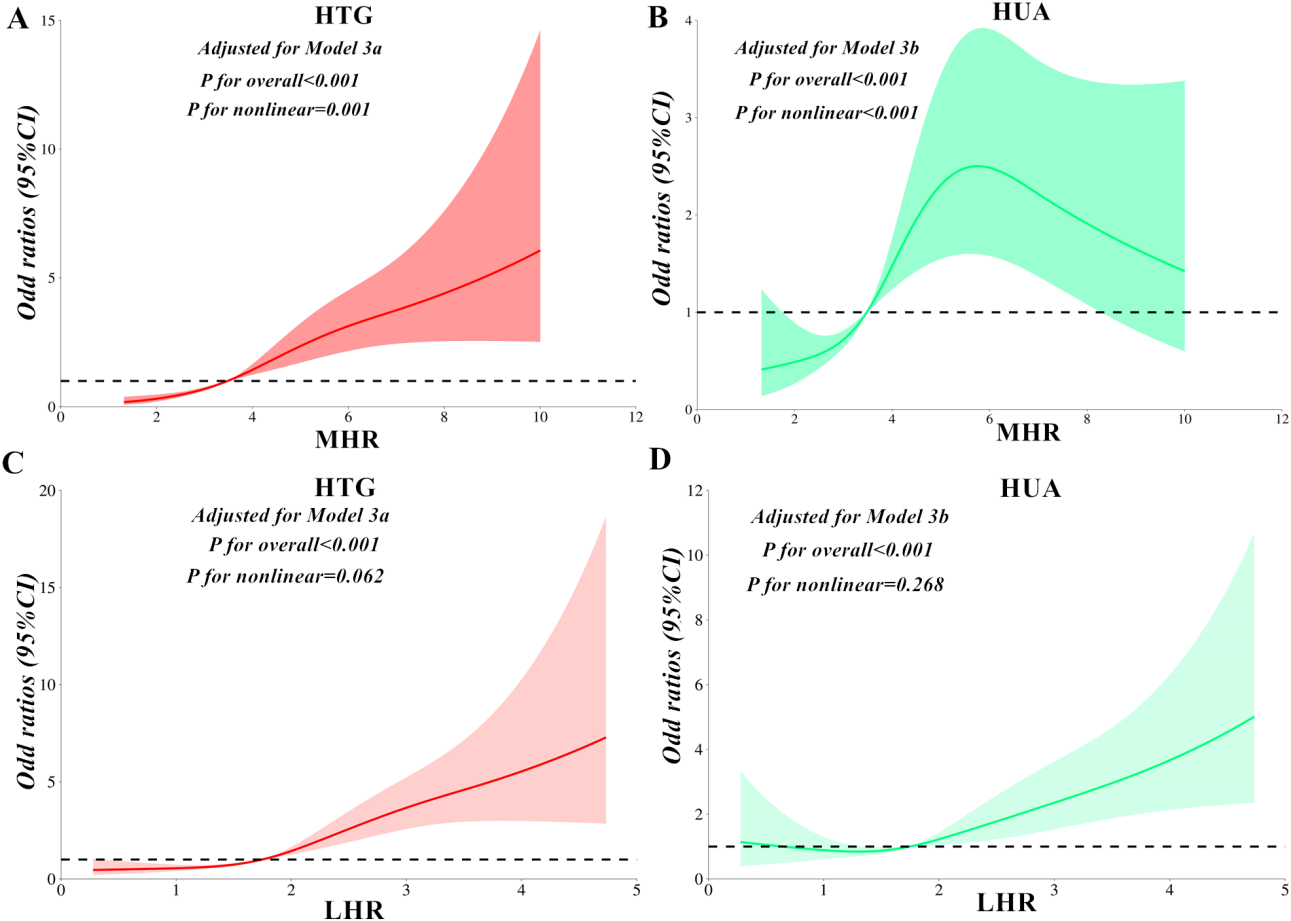
Restricted cubic spline analysis for the correlations of MHR **(A, B)** and LHR **(C, D)** with HTG and HUA after adjusting for Model 3^a^ or Model 3^b^. MHR, monocytes to high-density lipoprotein cholesterol ratio; LHR, lymphocytes to high-density lipoprotein cholesterol ratio; HTG, hypertriglyceridemia; HUA, hyperuricemia.

**Table 2** presents the results of binomial logistic regression analysis examining the correlations of MHR with HTG and HUA. The results showed that MHR displayed significantly elevated risks of both conditions in the third and fourth quartiles than the first quartile in all three models (All *P < 0.05*). Meanwhile, MHR exhibited independent correlations with HTG (**Figure 2A**) and HUA (**Figure 2B**) after applying full adjustments in Model 3^a^ or Model 3^b^. Specifically, the *ORs (95% CI)* were 1.59 (1.43-1.77) for HTG and 1.13 (1.04-1.23) for HUA (both *P < 0.05*). Additionally, each standard deviation (SD) increase in MHR contributed to an elevated risk of HTG (*OR:*2.60*, 95%CI:* 2.08-3.25*; P < 0.001*) in Model 3^a^ and HUA (*OR:*1.29*, 95%CI:* 1.08-1.54*; P =* 0.005) in Model 3^a^. As illustrated in **Figures 3A, B**, MHR exhibited a nonlinear relationship with both HTG (*P for nonlinear = 0.001*) and HUA (*P for nonlinear < 0.001*) in the RCS analysis. Furthermore, as shown in **Table 3**, a significant threshold effect was identified between MHR, HTG, and HUA (*P for log-likelihood ratio < 0.001*). Specifically, there were positive correlations of MHR with HTG and HUA observed when MHR values were below 5.24 and 4.76, respectively. The *ORs (95% CI)* were 2.04 (1.69-2.46) for HTG and 1.95 (1.44-2.66) for HUA (both *P < 0.001*). Conversely, no significant associations were observed when MHR exceeded the thresholds of 5.24 and 4.76.

**Table 2:** Binomial logistic regression analysis for the correlations of MHR with HTG and HUA.

Variable	Model 1		Model 2		Model 3^a^/3^b^	
	*OR (95% CI)*	*P* value	*OR (95% CI)*	*P* value	*OR (95% CI)*	*P* value
HTG						
Per SD increase	1.84 (1.52-2.21)	<0.001	2.67 (2.14-3.33)	<0.001	2.60 (2.08-3.25)	<0.001
Q1	Ref. (1.0)		Ref. (1.0)		Ref. (1.0)	
Q2	1.77 (1.19-2.62)	0.005	2.30 (1.49-3.54)	<0.001	2.29 (1.49-3.53)	<0.001
Q3	2.68 (1.81-3.97)	<0.001	4.12 (2.65-6.39)	<0.001	3.98 (2.56-6.19)	<0.001
Q4	4.02 (2.69-6.03)	<0.001	8.06 (5.00-12.99)	<0.001	7.69 (4.75-12.44)	<0.001
HUA						
Per SD increase	1.33 (1.13-1.56)	<0.001	1.36 (1.14-1.62)	<0.001	1.29 (1.08-1.54)	0.005
Q1	Ref. (1.0)		Ref. (1.0)		Ref. (1.0)	
Q2	1.05 (0.58-1.90)	0.878	1.16 (0.61-2.20)	0.647	1.11 (0.58-2.10)	0.758
Q3	2.20 (1.28-3.79)	0.004	2.79 (1.54-5.02)	<0.001	2.52 (1.38-4.59)	0.002
Q4	3.01 (1.77-5.12)	<0.001	3.84 (2.14-6.89)	<0.001	3.40 (1.86-6.22)	<0.001

Model 1 adjusted for age, diabetic duration, sex, alcohol consumption (yes or no), smoking status (yes or no), hypertension status (yes or no), body mass index, and waist circumference. Model 2 was further adjusted for glycated hemoglobin, total cholesterol, low-density lipoprotein cholesterol, alanine aminotransferase, aspartate aminotransferase, albumin, blood urea nitrogen, creatinine, and homeostasis model assessment insulin resistance based on Model 1. Model 3^a^ was further adjusted for HUA based on Model 2. Model 3^b^ was further adjusted for HTG based on Model 2. MHR, monocytes to high-density lipoprotein cholesterol ratio; HTG, hypertriglyceridemia; HUA, hyperuricemia.

**Table 3:** Threshold effect analysis of MHR on HTG and HUA using an adjusted two-piecewise logistic regression.

Characteristic	Adjusted OR (95% CI)	*P value*
HTG (Adjusted for Model 3^a^)		
Infection point	5.24	
<5.24	2.04 (1.69-2.46)	<0.001
≥5.24	1.12 (0.84-1.50)	0.437
P for likelihood test		<0.001
HUA (Adjusted for Model 3^b^)		
Infection point	4.76	
<4.76	1.95 (1.44-2.66)	<0.001
≥4.76	0.91 (0.72-1.15)	0.415
*P* for likelihood test		<0.001

Model 3^a^ adjusted for age, diabetic duration, sex, alcohol consumption (yes or no), smoking status (yes or no), hypertension status (yes or no), body mass index, waist circumference, glycated hemoglobin, total cholesterol, low-density lipoprotein cholesterol, alanine aminotransferase, aspartate aminotransferase, albumin, blood urea nitrogen, creatinine, homeostasis model assessment insulin resistance, and HUA. Model 3^b^ adjusted for age, diabetic duration, sex, alcohol consumption, smoking, hypertension, body mass index, waist circumference, glycated hemoglobin, total cholesterol, low-density lipoprotein cholesterol, alanine aminotransferase, aspartate aminotransferase, albumin, blood urea nitrogen, creatinine, homeostasis model assessment insulin resistance, and HTG. MHR, monocytes to high-density lipoprotein cholesterol ratio; HTG, hypertriglyceridemia; HUA, hyperuricemia.

### Correlation of LHR with the risk of HTG and HUA

Following full adjustments in Model 3^a^ or Model 3^b^, LHR exhibited significantly higher risks of HTG and HUA in the fourth quartile than the first quartile (**Table 4**) (Both *P < 0.001*). LHR was independently correlated with HTG (**Figure 2C**) and HUA (**Figure 2D**), with *ORs (95% CI)* of 2.34 (1.91-2.88) and 1.63 (1.33-2.00), respectively. Each SD increase in LHR contributed to an elevated risk of HTG (*OR:*2.18*, 95%CI:*1.80-2.63*; P < 0.001*) and HUA (*OR:*1.56*, 95%CI:* 1.30-1.88*; P < 0.001*). Conversely, a linear correlation of LHR with HTG (*P for nonlinear = 0.062*) and HUA (*P for nonlinear = 0.268*) (**Figures 3C, D**) was observed in the RCS analysis. Meanwhile, there were no significant threshold effects observed between LHR, HTG, and HUA (*P for log-likelihood ratio > 0.05*).

**Table 4:** Binomial logistic regression analysis for the correlations of LHR with HTG and HUA.

Variable	Model 1		Model 2		Model 3^a^/3^b^	
	*OR (95% CI)*	*P* value	*OR (95% CI)*	*P* value	*OR (95% CI)*	*P* value
HTG						
Per SD increase	1.68 (1.44-1.96)	<0.001	2.22 (1.84-2.67)	<0.001	2.18 (1.80-2.63)	<0.001
Q1	Ref. (1.0)		Ref. (1.0)		Ref. (1.0)	
Q2	1.30 (0.88-1.90)	0.186	1.78 (1.17-2.70)	0.007	1.75 (1.15-2.66)	0.009
Q3	1.62 (1.10-2.37)	0.014	2.84 (1.84-4.37)	<0.001	2.82 (1.83-4.34)	<0.001
Q4	3.64 (2.46-5.39)	<0.001	7.61 (4.79-12.14)	<0.001	7.22 (4.50-11.57)	<0.001
HUA						
Per SD increase	1.54 (1.31-1.81)	<0.001	1.64 (1.37-1.96)	<0.001	1.56 (1.30-1.88)	<0.001
Q1	Ref. (1.0)		Ref. (1.0)		Ref. (1.0)	
Q2	1.30 (0.76-2.24)	0.338	1.37 (0.76-2.46)	0.294	1.31 (0.72-2.36)	0.724
Q3	1.14 (0.66-1.97)	0.628	1.32 (0.73-2.37)	0.355	1.22 (0.67-2.20)	0.514
Q4	2.87 (1.75-4.71)	<0.001	3.66 (2.11-6.35)	<0.001	3.17 (1.79-5.61)	<0.001

Model 1 adjusted for age, diabetic duration, sex, alcohol consumption (yes or no), smoking status (yes or no), hypertension status (yes or no), body mass index, and waist circumference. Model 2 was further adjusted for glycated hemoglobin, total cholesterol, low-density lipoprotein cholesterol, alanine aminotransferase, aspartate aminotransferase, albumin, blood urea nitrogen, creatinine, and homeostasis model assessment insulin resistance based on Model 1. Model 3^a^ was further adjusted for HUA based on Model 2. Model 3^b^ was further adjusted for HTG based on Model 2. LHR, lymphocytes to high-density lipoprotein cholesterol ratio; HTG, hypertriglyceridemia; HUA, hyperuricemia.

### MHR and LHR mediate the impact of HTG on HUA

**Figure 4:**
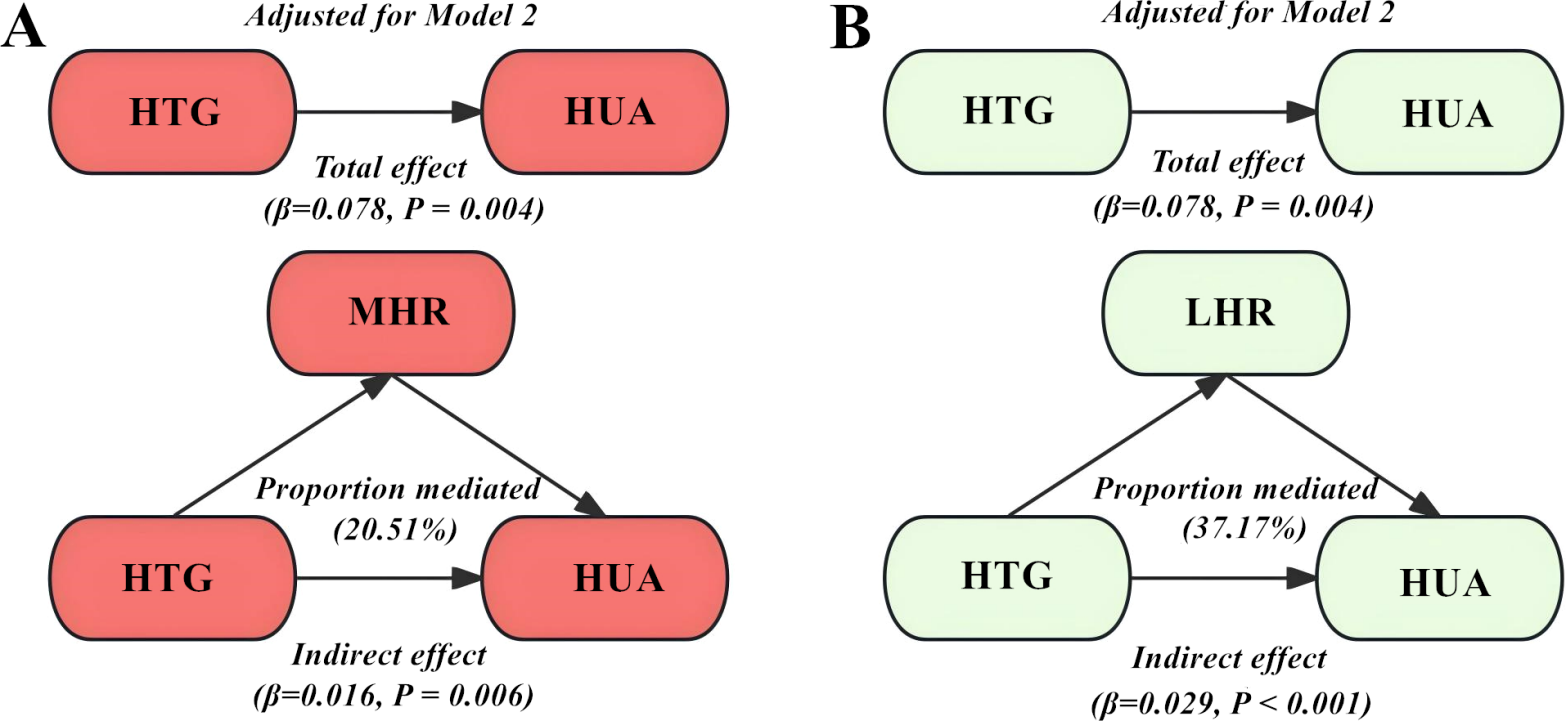
Structural models represent the mediating effect of MHR **(A)** and LHR **(B)** in the impact of HTG on HUA using the adjusted mediation analysis. MHR, monocytes to high-density lipoprotein cholesterol ratio; LHR, lymphocytes to high-density lipoprotein cholesterol ratio; HTG, hypertriglyceridemia; HUA, hyperuricemia.

The adjusted mediation analysis exhibited that MHR and LHR partially mediated the impact of HTG on HUA in both Model 1 and Model 2 (**Figure 4**). After further applying the adjustment in Model 2, the total effect of HTG on HUA was 0.078 (*P* = 0.004), the indirect effect of MHR and LHR on this association was 0.016 (*P* = 0.006) and 0.029 (*P < 0.001*), accounting for the mediated proportion of 20.51% and 37.17%, respectively.

### Sensitive analysis

**Figure 5:**
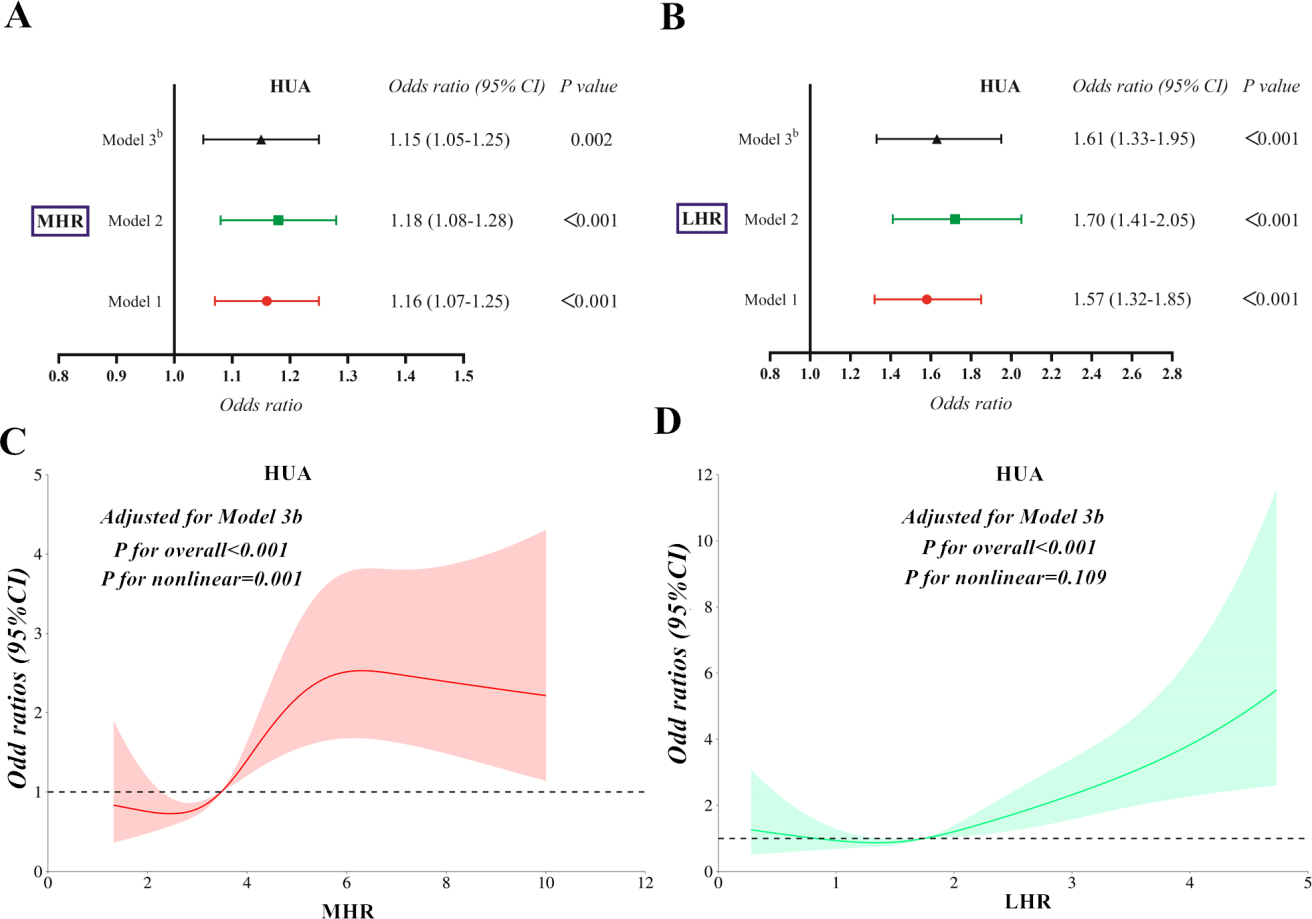
Binomial logistic regression **(A, B)** and restricted cubic spline **(C, D)** analysis for the correlations of MHR and LHR with HUA in the sensitivity analysis. MHR, monocytes to high-density lipoprotein cholesterol ratio; LHR, lymphocytes to high-density lipoprotein cholesterol ratio; HTG, hypertriglyceridemia; HUA, hyperuricemia.

The sensitivity analysis yielded consistent results when applying the previous diagnostic criteria for HUA. A total of 222 (22.7%) participants were diagnosed with HUA. Following the adjustment for Model 3^b^, HUA remained significantly associated with MHR (*OR:*1.15*, 95%CI:* 1.05-1.25*; P =* 0.002) (**Figure 5A**) and LHR (*OR:*1.61*, 95%CI:* 1.33-1.95*; P < 0.001*) (**Figure 5B**). Moreover, HUA remained a nonlinear association with MHR (*P for nonlinear = 0.001*) (**Figure 5C**) and persisted as a linear correlation with LHR (*P for nonlinear = 0.109*) (**Figure 5D**). Furthermore, the mediation effects of MHR and LHR on the association between HTG and HUA persisted. The total effect of HTG on HUA was 0.093 (*P < 0.001*), the indirect effect of MHR and LHR on this association was 0.022 (*P* = 0.002) and 0.036 (*P < 0.001*), with mediated proportions of 23.66% and 38.70%, respectively, after applying the adjustments in Model 2.

## Discussion

HUA is a common metabolic disorder associated with significant comorbidities, representing a substantial global public health burden. Recent research indicates that HTG may facilitate the onset of HUA by inducing systemic inflammation and oxidative stress. The underlying mechanisms are believed to involve various immune cells (eg, monocytes and lymphocytes) and the dysregulation of HDL-c. As markers of inflammation and oxidative stress, MHR and LHR have been extensively utilized in previous studies. This study investigates the roles of MHR and LHR in the impact of HTG on HUA in DM, thereby enhancing our understanding of the contributions of inflammation and oxidative stress within this complex interplay. The results exhibited that both the MHR and LHR exhibited a significant association with HUA and HTG, as determined by binomial logistic regression and RCS analysis. Notably, MHR and LHR also partially mediate the impact of HTG on HUA. Moreover, Sensitivity analyses confirmed these findings when applying previous diagnostic criteria for HUA, reinforcing the robustness of the results.

Oxidative stress initiates a cascade of pathological processes that concurrently activate inflammatory pathways, ultimately establishing a vicious cycle of inflammation and oxidative stress. This interplay is crucial in the development and progression of metabolic disorders, such as DM, HUA, HTG, and hypertension (6, 24, 25). Furthermore, these metabolic disorders can exacerbate inflammation and oxidative stress, perpetuating this cycle. HTG is the most common metabolic disorder that can result in a reduction in antioxidant production while simultaneously promoting an increase in pro-oxidant levels. This imbalance between antioxidants and oxidants can trigger an inflammatory response. Collectively, these processes can amplify inflammation and oxidative stress, highlighting their role in the pathogenesis of HTG and their contribution to other metabolic disorders, such as HUA. Immune cells are integral to this dynamic, significantly contributing to modulating the severity and progression of inflammatory processes and oxidative stress. The infiltration of immune cells along with the excessive expression of inflammatory cytokines by these cells plays a pivotal role in the process of inflammatory response and oxidative stress (26, 27). In particular, monocytes and lymphocytes play crucial roles, as they are activated by inflammatory factors such as tumor necrosis factor-alpha. This activation involves the translocation of nuclear factor-kappa B and activator protein-1 into the nucleus. As a result, the macrophages can produce substantial amounts of reactive nitrogen species, reactive oxygen species, and peroxynitrite by a mechanism known as the respiratory burst (28). These processes may lead to epigenetic modifications that contribute to the pathogenesis of metabolic diseases (29).

Emerging evidence demonstrated that HUA exhibited characteristics of inflammatory and immune system disorders, thus rendering it more than just a simple metabolic disease. In the context of advanced gout, monosodium urate crystal deposition can regulate monocyte populations, increasing classical monocytes (30). Similarly, T lymphocytes exhibit elevated expression of inflammatory factors due to direct stimulation by T-cell receptors, such as CD25, CD80, and CD86, in a high UA environment (14, 31). Our findings further underscore the critical roles of these immune cells in HUA and HTG, as participants in the HUA and HTG groups demonstrated significantly elevated levels of both monocytes and lymphocytes. HDL-c possesses multiple metabolic protective functions, including the facilitation of cholesterol excretion from cells. Beyond this crucial role, HDL-c also exhibits antioxidant, anti-inflammatory, anti-apoptotic, and anti-thrombotic effects, all of which contribute significantly to cardiovascular health and the overall regulation of metabolic processes. Increasing evidence indicates that inflammation and oxidative stress can alter the quantity and quality of HDL, leading to reduced HDL levels and abnormal function (17, 32, 33). Metabolic disorders are often accompanied by decreased HDL-c levels, and our findings further reinforce this observation in HUA and HTG, as participants in these groups demonstrated significantly decreased levels of HDL-c. This relationship highlights the interconnectedness of lipid metabolism, immune response, and inflammatory processes in the pathogenesis of these conditions.

The roles of monocytes, lymphocytes, and HDL-c in inflammation and oxidative stress have led to the establishment of derived biomarkers, namely MHR and LHR. These ratios are increasingly recognized for their associations with various cardiometabolic diseases. For instance, Uslu et al. identified that MHR exhibited independent associations with the prevalence and severity of metabolic syndrome (34). Our previous research demonstrated that MHR can be used as an indicator for metabolic syndrome in T2DM (35). Similarly, Studies by Chen et al. and Yu et al. found that LHR was independently correlated with metabolic syndrome, with a predictive value for this condition (19, 36). Furthermore, elevated values of MHR and LHR have been shown to independently correlate with an increased risk of cardiovascular-kidney-metabolic syndrome across stages 1 to 4 (37). Additionally, previous research has established a significant correlation of MHR with HUA and UA. Chen et al. found that increased MHR demonstrated a positive correlation with the presence of HUA in a cohort of rural Chinese adults (38). Moreover, Huang et al. revealed that higher MHR contributed to an elevated risk of HUA in a cohort of T2DM, and BMI played a key mediating role in this relationship (39). Li et al. revealed a positive correlation of MHR with UA after adjusting for confounders in 646 participants (40). Consistent with these findings, the present study also demonstrates a significant correlation of MHR with HUA in a cohort of DM. While our research has established an independent association between LHR and HUA, this relationship requires further validation through additional studies. Consequently, more comprehensive investigations are necessary to elucidate this association. Previous research has also identified significant correlations between MHR, LHR, and HTG. Hashemi et al. found a positive association between LHR and TG levels (41), while Cândido et al. revealed that LHR levels were independently associated with HTG in secondary care settings (42). Studies by Xi et al. also revealed a positive correlation of MHR with TG in 3848 physical examiners (43). Based on these findings, both LHR and MHR emerge as potential predictors of HTG and HUA, highlighting that inflammation and oxidative stress may contribute to a crucial role in the pathogenesis of these two conditions. It is important to highlight that the associations found between LHR, MHR, HTG, and HUA in this study, as well as in previous research, largely stem from cross-sectional observational studies. This design can limit the ability to draw causal inferences. Future studies utilizing longitudinal or experimental designs may be necessary to better understand the nature of these relationships and establish causality. A notable observation from this study is that MHR and LHR partially mediate the impact of HTG on HUA, even after adjusting for potential confounding factors in the mediation analysis. Sensitivity analyses yielded consistent results when applying previously established diagnostic criteria for HUA, suggesting that the inflammation and oxidative stress reflected by these biomarkers may contribute to the association between HTG and HUA.

### Strength and limitation

The strengths of this study lie in its comprehensive examination of the mediating roles of MHR and LHR in the impact of HTG on HUA. Furthermore, the application of the other diagnostic criteria for HUA through sensitivity analysis enhances the robustness and validity of the findings. However, several limitations warrant consideration. A primary limitation is the study’s reliance on observational data, which may be susceptible to confounding factors that were not adequately controlled, particularly concerning dietary habits and physical activity levels. Additionally, the study cohort consisted solely of individuals with DM from a single center, raising concerns about the generalizability of the findings to broader populations and diverse ethnic groups. To address these limitations, future studies are essential to evaluate the applicability of these results beyond a single center, and including varied populations will help validate the findings and enhance their relevance in clinical practice.

## Conclusion

In conclusion, MHR and LHR were independently associated with HTG and HUA in DM. Furthermore, both MHR and LHR partially mediated the impact of HTG on HUA. These findings may indicate the underlying mechanisms involving inflammation and oxidative stress that may contribute to the impact of HTG on HUA from a clinical perspective. This insight may inform future interventions aimed at mitigating the risks associated with these conditions.

## Data Availability

The raw data supporting the conclusions of this article will be made available by the authors, without undue reservation.

## Supplementary material

Supplementary Figure 1 Flowchart illustrating the selection and exclusion process of the study cohort. DM, diabetes mellitus; UA, uric acid; TG, triglyceride.

## References

[1] Kang S Han K Jung J Eun Y Kim IY Koh EM . Women with metabolic syndrome and unhealthy lifestyle factors are at a higher risk for hyperuricemia. J Clin Med. (2023) 12:7159. doi: 10.3390/jcm12227159. PMID: 38002772 PMC10671870

[2] Qin T Zhou X Wang J Wu X Li Y Wang L . Hyperuricemia and the prognosis of hypertensive patients: a systematic review and meta-analysis. J Clin Htn. (2016) 18:1268–78. doi: 10.1111/jch.12855. PMID: 27247021 PMC8031654

[3] Toda A Ishizaka Y Tani M Yamakado M . Hyperuricemia is a significant risk factor for the onset of chronic kidney disease. Nephron Clin Pract. (2014) 126:33–8. doi: 10.1159/000355639. PMID: 24434843

[4] Lyu D Zhuang R Li J Wu Y Di Y Song M . Association of hyperuricemia with coronary heart disease and other cardiovascular outcomes: a systematic review and dose-response meta-analysis. PloS One. (2025) 20:e0337091. doi: 10.1371/journal.pone.0337091. PMID: 41252397 PMC12626327

[5] Jiang J Zhang T Liu Y Chang Q Zhao Y Guo C . Prevalence of diabetes in patients with hyperuricemia and gout: a systematic review and meta-analysis. Curr Diabetes Rep. (2023) 23:103–17. doi: 10.1007/s11892-023-01506-2. PMID: 37099085

[6] Xu L Li C Wan T Sun X Lin X Yan D . Targeting uric acid: a promising intervention against oxidative stress and neuroinflammation in neurodegenerative diseases. Cell Commun Signaling. (2025) 23:4. doi: 10.1186/s12964-024-01965-4. PMID: 39754256 PMC11699683

[7] Ghaemi-Oskouie F Shi Y . The role of uric acid as an endogenous danger signal in immunity and inflammation. Curr Rheumatol Rep. (2011) 13:160–6. doi: 10.1007/s11926-011-0162-1. PMID: 21234729 PMC3093438

[8] Lu J Wang Z Mei W Peng K Zhang L Wang G . A systematic review of the epidemiology and risk factors for severity and recurrence of hypertriglyceridemia-induced acute pancreatitis. BMC Gastroenterol. (2025) 25:374. doi: 10.1186/s12876-025-03954-4. PMID: 40375154 PMC12082898

[9] Nordestgaard BG Varbo A . Triglycerides and cardiovascular disease. Lancet. (2014) 384:626–35. doi: 10.1016/s0140-6736(14)61177-6. PMID: 25131982

[10] Hou YL Yang XL Wang CX Zhi LX Yang MJ You CG . Hypertriglyceridemia and hyperuricemia: a retrospective study of urban residents. Lipids Health Dis. (2019) 18:81. doi: 10.1186/s12944-019-1031-6. PMID: 30935401 PMC6444567

[11] Chen JH Pan WH Hsu CC Yeh WT Chuang SY Chen PY . Impact of obesity and hypertriglyceridemia on gout development with or without hyperuricemia: a prospective study. Arthritis Care Res. (2013) 65:133–40. doi: 10.1002/acr.21824. PMID: 22933424

[12] Fox IH John D DeBruyne S Dwosh I Marliss EB . Hyperuricemia and hypertriglyceridemia: metabolic basis for the association. Metabolism. (1985) 34:741–6. doi: 10.1016/0026-0495(85)90025-3. PMID: 4021806

[13] Yingchun H Yahong M Jiangping W Xiaokui H Xiaohong Z . Increased inflammation, endoplasmic reticulum stress and oxidative stress in endothelial and macrophage cells exacerbate atherosclerosis in ApoCIII transgenic mice. Lipids Health Dis. (2018) 17:220. doi: 10.1186/s12944-018-0867-5. PMID: 30223835 PMC6142424

[14] Webb R Jeffries M Sawalha AH . Uric acid directly promotes human T-cell activation. Am J Med Sci. (2009) 337:23–7. doi: 10.1097/maj.0b013e31817727af. PMID: 19057377

[15] Wu Z Zhao L Guo Y Lin C Lu P He Q . Hyperuricemia exacerbates experimental periodontitis via uric acid-induced periodontal inflammation and oxidative stress. J Clin Periodontol. (2025) 52:773–86. doi: 10.1111/jcpe.14144. PMID: 39976076

[16] Li D Yuan S Deng Y Wang X Wu S Chen X . The dysregulation of immune cells induced by uric acid: mechanisms of inflammation associated with hyperuricemia and its complications. Front Immunol. (2023) 14:1282890. doi: 10.3389/fimmu.2023.1282890. PMID: 38053999 PMC10694226

[17] Tabet F Rye KA . High-density lipoproteins, inflammation and oxidative stress. Clin Sci. (2009) 116:87–98. doi: 10.1042/cs20080106. PMID: 19076062

[18] Ganjali S Gotto AM Jr Ruscica M Atkin SL Butler AE Banach M . Monocyte-to-HDL-cholesterol ratio as a prognostic marker in cardiovascular diseases. J Cell Physiol. (2018) 233:9237–46. doi: 10.1002/jcp.27028. PMID: 30076716

[19] Yu S Guo X Li G Yang H Zheng L Sun Y . Lymphocyte to high-density lipoprotein ratio but not platelet to lymphocyte ratio effectively predicts metabolic syndrome among subjects from rural China. Front Cardiovasc Med. (2021) 8:583320. doi: 10.3389/fcvm.2021.583320. PMID: 33778016 PMC7994280

[20] Diabetes ADAPPCf . 2. Diagnosis and classification of diabetes: standards of care in diabetes-2026. Diabetes Care. (2026) 49:S27–49. doi: 10.2337/dc26-s002. PMID: 41358893 PMC12690183

[21] Berglund L Brunzell JD Goldberg AC Goldberg IJ Sacks F Murad MH . Evaluation and treatment of hypertriglyceridemia: an Endocrine Society clinical practice guideline. J Clin Endocrinol Metab. (2012) 97:2969–89. doi: 10.1210/jc.2011-3213. PMID: 22962670 PMC3431581

[22] Sun M Lyu Z Wang C Li Y Zhao D Ran X . 2024 update of Chinese guidelines for diagnosis and treatment of hyperuricemia and gout part I: recommendations for general patients. Int J Rheumatic Dis. (2025) 28:e70375. doi: 10.1111/1756-185x.70375. PMID: 40692263 PMC12280528

[23] Li Q Li X Wang J Liu H Kwong JS Chen H . Diagnosis and treatment for hyperuricemia and gout: a systematic review of clinical practice guidelines and consensus statements. BMJ Open. (2019) 9:e026677. doi: 10.1136/bmjopen-2018-026677. PMID: 31446403 PMC6720466

[24] Masenga SK Kabwe LS Chakulya M Kirabo A . Mechanisms of oxidative stress in metabolic syndrome. Int J Mol Sci. (2023) 24:7898. doi: 10.3390/ijms24097898. PMID: 37175603 PMC10178199

[25] Yaribeygi H Sathyapalan T Atkin SL Sahebkar A . Molecular mechanisms linking oxidative stress and diabetes mellitus. Oxid Med Cell Longevity. (2020) 2020:8609213. doi: 10.1155/2020/8609213. PMID: 32215179 PMC7085395

[26] Chang JG Tu SJ Huang CM Chen YC Chiang HS Lee YT . Single-cell RNA sequencing of immune cells in patients with acute gout. Sci Rep. (2022) 12:22130. doi: 10.1038/s41598-022-25871-2. PMID: 36550178 PMC9772586

[27] Xu H Zhang B Chen Y Zeng F Wang W Chen Z . Type II collagen facilitates gouty arthritis by regulating MSU crystallization and inflammatory cell recruitment. Ann Rheumatic Dis. (2023) 82:416–27. doi: 10.1136/ard-2022-222764. PMID: 36109143

[28] Castaneda OA Lee SC Ho CT Huang TC . Macrophages in oxidative stress and models to evaluate the antioxidant function of dietary natural compounds. J Food Drug Anal. (2017) 25:111–8. doi: 10.1016/j.jfda.2016.11.006. PMID: 28911528 PMC9333431

[29] Saitoh S Van Wijk K Nakajima O . Crosstalk between metabolic disorders and immune cells. Int J Mol Sci. (2021) 22:10017. doi: 10.3390/ijms221810017. PMID: 34576181 PMC8469678

[30] Gu H Yu H Qin L Yu H Song Y Chen G . MSU crystal deposition contributes to inflammation and immune responses in gout remission. Cell Rep. (2023) 42:113139. doi: 10.1016/j.celrep.2023.113139. PMID: 37756161

[31] Yamaoka T Yano M Kondo M Sasaki H Hino S Katashima R . Feedback inhibition of amidophosphoribosyltransferase regulates the rate of cell growth via purine nucleotide, DNA, and protein syntheses. J Biol Chem. (2001) 276:21285–91. doi: 10.1074/jbc.m011103200. PMID: 11290738

[32] Wilson PG Thompson JC Shridas P McNamara PJ de Beer MC de Beer FC . Serum amyloid A is an exchangeable apolipoprotein. Arteriosclerosis Thrombosis Vasc Biol. (2018) 38:1890–900. doi: 10.1161/atvbaha.118.310979. PMID: 29976766 PMC6202200

[33] Brites F Martin M Guillas I Kontush A . Antioxidative activity of high-density lipoprotein (HDL): mechanistic insights into potential clinical benefit. BBA Clin. (2017) 8:66–77. doi: 10.1016/j.bbacli.2017.07.002. PMID: 28936395 PMC5597817

[34] Uslu AU Sekin Y Tarhan G Canakcı N Gunduz M Karagulle M . Evaluation of monocyte to high-density lipoprotein cholesterol ratio in the presence and severity of metabolic syndrome. Clin Appl Thromb Hemostasis. (2018) 24:828–33. doi: 10.1177/1076029617741362. PMID: 29212375 PMC6714883

[35] Wang W Chen ZY Guo XL Tu M . Monocyte to high-density lipoprotein and apolipoprotein A1 ratios: novel indicators for metabolic syndrome in Chinese newly diagnosed type 2 diabetes. Front Endocrinol. (2022) 13:935776. doi: 10.3389/fendo.2022.935776. PMID: 35909551 PMC9330493

[36] Chen H Xiong C Shao X Ning J Gao P Xiao H . Lymphocyte to high-density lipoprotein ratio as a new indicator of inflammation and metabolic syndrome. Diabetes Metab Syndrome Obesit: Targets Ther. (2019) 12:2117–23. doi: 10.2147/dmso.s219363. PMID: 31686883 PMC6798814

[37] Song J Xu Z Yu H Li A Liu Y Jin M . Association of three composite inflammatory and lipid metabolism indicators with cardiovascular-kidney-metabolic syndrome: a cross-sectional study based on NHANES 1999-2020. Mediators Inflammation. (2025) 2025:6691516. doi: 10.1155/mi/6691516. PMID: 40376312 PMC12081155

[38] Chen MQ Shi WR Shi CN Zhou YP Sun YX . Impact of monocyte to high-density lipoprotein ratio on prevalent hyperuricemia: findings from a rural Chinese population. Lipids Health Dis. (2020) 19:48. doi: 10.1186/s12944-020-01226-6. PMID: 32178680 PMC7077021

[39] Huang B Li X Zhang XX Li SW Wang M Chen Q . MHR was associated with hyperuricemia risk in patients with type 2 diabetes mellitus: the mediating effect of body mass index. Diabetes Metab Syndrome Obesit: Targets Ther. (2025) 18:3015–25. doi: 10.2147/dmso.s535669. PMID: 40874092 PMC12379993

[40] Li Y Liu X Luo Y . Monocyte to high-density lipoprotein cholesterol ratio and serum uric acid in Chinese adults: a cross-sectional study. BMC Endocr Disord. (2022) 22:48. doi: 10.1186/s12902-022-00966-z. PMID: 35216583 PMC8881867

[41] Hashemi SM Kheirandish M Rafati S Ghazalgoo A Amini-Salehi E Keivanlou MH . The association between neutrophil and lymphocyte to high-density lipoprotein cholesterol ratio and metabolic syndrome among Iranian population, finding from Bandare Kong cohort study. Lipids Health Dis. (2024) 23:393. doi: 10.1186/s12944-024-02378-5. PMID: 39604922 PMC11603836

[42] Cândido FG da Silva A Zanirate GA Oliveira N Hermsdorff HHM . Lymphocyte to high-density lipoprotein cholesterol ratio is positively associated with pre-diabetes, metabolic syndrome, and non-traditional cardiometabolic risk markers: a cross-sectional study at secondary health care. Inflammation. (2025) 48:276–87. doi: 10.1007/s10753-024-02063-w 38844648

[43] Xi J Men S Nan J Yang Q Dong J . The blood monocyte to high density lipoprotein cholesterol ratio (MHR) is a possible marker of carotid artery plaque. Lipids Health Dis. (2022) 21:130. doi: 10.1186/s12944-022-01741-8. PMID: 36463176 PMC9719628

