# Supplementary figures and images for: Immune cells and high-density lipoprotein cholesterol derivative markers mediate the impact of hypertriglyceridemia on hyperuricemia in diabetes mellitus
Source: Front Immunol. 2026 May 5;17:1816335. doi: 10.3389/fimmu.2026.1816335 (PMC13183575; doi:10.3389/fimmu.2026.1816335)

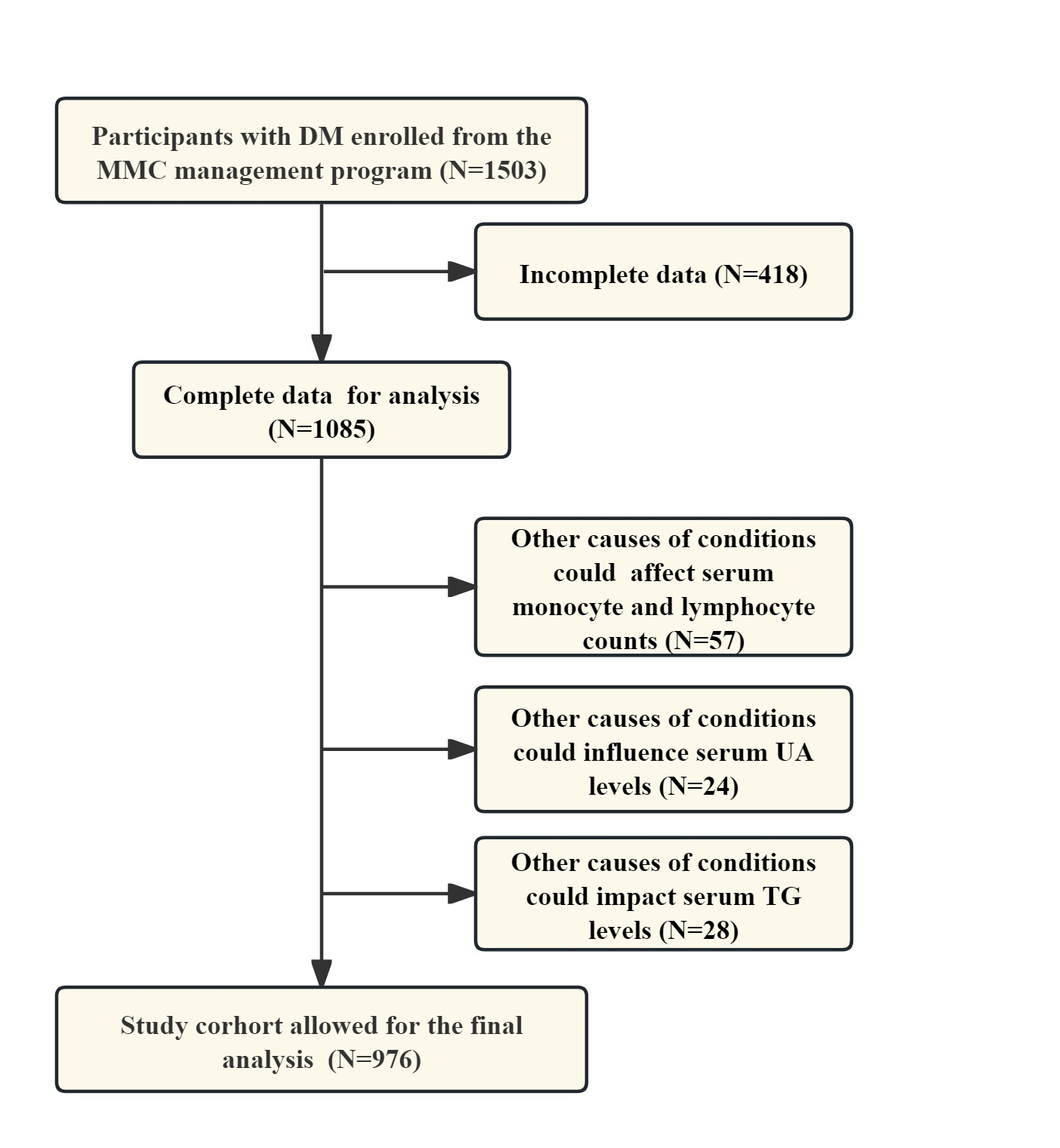

Supplement: Supplementary Figure 1 — Flowchart illustrating the selection and exclusion process of the study cohort. DM, diabetes mellitus; UA, uric acid; TG, triglyceride. [file Image1.jpeg]
